# Treatment of relapsing multiple sclerosis in Hungary – consensus recommendation from the Hungarian neuroimmunology society

**DOI:** 10.1186/s13023-023-02789-0

**Published:** 2023-07-07

**Authors:** Cecilia Rajda, Csilla Rózsa, Andrea Mike, Gábor Lovas, Zsolt Mezei, Gábor Jakab, Péter Ács, Gábor Rum, Magdolna Simó, Zita Jobbágy, Zita Bíró, Anita Trauninger, Piroska Imre, Klotild Mátyás, István Deme, Zsolt Illés, Tunde Csepany

**Affiliations:** 1 Department of Neurology, Albert Szent-Györgyi Health Centre, University of Szeged, Semmelweis u.6, Szeged, 6725 Hungary; 2 Department of Neurology, Jahn Ferenc Teaching Hospital, Köves u. 1, Budapest, 1204 Hungary; 3 Department of Neurology, Szent Borbála Hospital, Dózsa György u. 77, Tatabánya, 2800 Hungary; 4 Department of Neurology, Faculty of Medicine, Semmelweis University, Balassa u. 6, Budapest, 1083 Hungary; 5 Department of Neurology, Uzsoki Hospital, Uzsoki u. 29-41, Budapest, 1145 Hungary; 6 Department of Neurology, Medical School, University of Pécs, Rét u. 2, Pécs, 7623 Hungary; 7 Petz Aladár Department of Neurology, County Teaching Hospital, Vasvári Pál u. 2-4, Győr, 9024 Hungary; 8 Department of Neurology, Bács-Kiskun County Teaching Hospital, Kecskemét, Nyíri u. 38, Kecskemét, 6000 Hungary; 9 Department of Neurology, Flór Ferenc Hospital, Semmelweis tér 1, Kistarcsa, 2143 Hungary; 10 Department of Neurology, Csolnoky Ferenc Hospital, Kórház u. 1, Veszprém, 8200 Hungary; 11 Department of Neurology, Markhot Ferenc Teaching Hospital, Knézich K. u. 1, Eger, 3300 Hungary; 12 Department of Neurology, Kaposi Mór Teaching Hospital, Tallián Gyula u 20-32, Kaposvár, 7400 Hungary; 13 Department of Neurology, Odense University Hospital, Institute of Clinical Research, University of Southern Denmark, Winslows vej 4, Odense, 5000 Denmark; 14 Department of Neurology, Faculty of Medicine, University of Debrecen, Móricz Zs. Krt. 22, Debrecen, 4032 Hungary

**Keywords:** Consensus, Multiple sclerosis, Recommendation, Treatment

## Abstract

Multiple sclerosis (MS) may impact quality of life, careers and family plans of the affected individuals. The current treatments with disease modifying therapies aim to prevent people with MS (pwMS) from disability accumulation and progression. Different countries have different reimbursement policies resulting in inequalities in patient care among geographical regions. Access to anti-CD20 therapies for relapsing MS is restricted in Hungary because therapy of individual cases only is reimbursed. In the light of the latest research and national guidelines, 17 Hungarian MS experts agreed on 8 recommendations regarding relapsing pwMS using the Delphi round method. Strong agreement (> 80%) was achieved in all except one recommendation after three rounds, which generated a fourth Delphi round. The experts agreed on treatment initiation, switch, follow-up and discontinuation, as well as on special issues such as pregnancy, lactation, elderly population, and vaccination. Well-defined national consensus protocols may facilitate dialogue between policymakers and healthcare professionals and thus contribute to better patient care in the long run.

**Supplementary Information:**

The online version contains supplementary material available at 10.1186/s13023-023-02789-0.

## Introduction

MS is a leading cause of disability among young adults. This chronic neurodegenerative and autoimmune disease affects patients in their productive and fertile years. MS may also have an impact on the quality of life, professional career and family planning. Cognitive and physical disability has a significant economic burden and in a worst-case scenario leads to social isolation. The prevalence of MS is rising worldwide. In Hungary, MS prevalence is estimated between 101.4-127.2/100,000 measured by two different methodological approaches [1, 2]. This is similar to the prevalence of the neighboring country Croatia (143.8/100,000) [3]. The worldwide incidence is also increasing, and the highest increase in both incidence and prevalence happened in the elderly populations, especially in women between the age of 50–60 years [4].

The current treatments with disease modifying therapies (DMTs) aim to prevent people with MS (pwMS) from disability accumulation and progression. The use of the current DMTs contributed to decreased prevalence of the late progressive course (secondary progression). The introduction of high-efficacy therapies (HET) early in the disease course may reduce, halt or even reverse disability progression [5, 6]. The difference between reimbursement policies in Denmark and Sweden led to differences in patient outcome measures favoring the start with HET over the escalation model, which starts with platform therapy and switches the treatment to higher efficacy drug when disease activity is shown [7]. Several other recent real-word studies worldwide observed similar advantages of treatment start with HET [8–10]. These data are shaping the treatment strategy of MS, moving from escalation to the early initiation with HET. Such novel strategies are reflected even in national treatment guidelines [11–13].

**Fig. 1:**
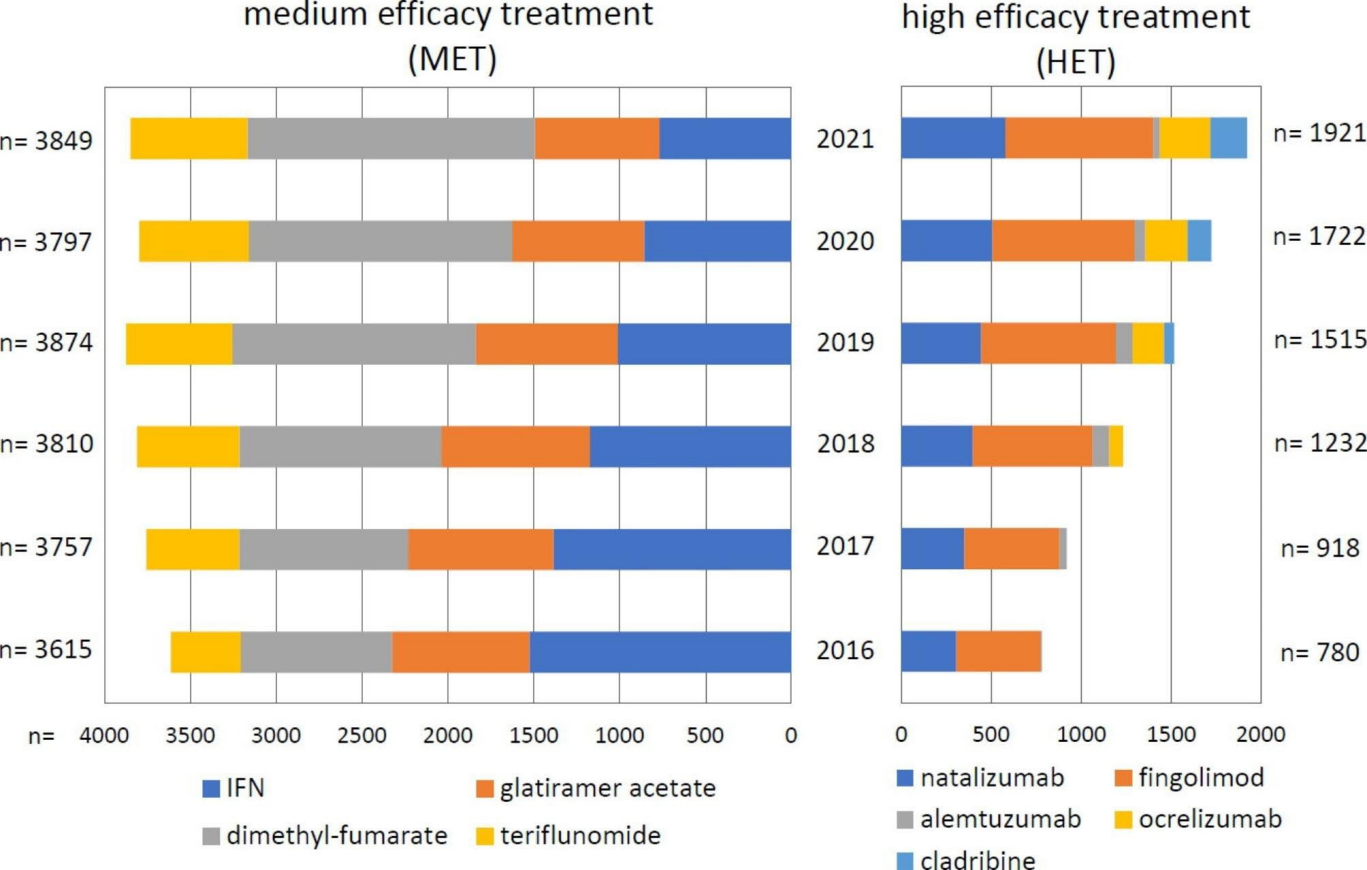
The number of medication prescription of pwMS on HET between 2016 and 2021 -based on the data from the National Health Insurance Fund [18]. The DMTs are shown in the order of their approval by the national authorities. Cladribine has been accepted since January 2020 without restriction. The reimbursement of anti-CD20 therapies are available with restriction, the only exception is ocrelizumab for primary progressive MS with unrestricted availability since January 2022. Data on DMTs n < 10, e.g. rituximab are not shown

The proportion of pwMS using HET has increased in Hungary as well along with markedly increased annual cost of MS treatments (Fig. 1). Of note, the clinical benefits and reduced disease burden are expected to be seen only in the long run. The measurement of socioeconomic advantages is even more complex.

Different countries have different reimbursement policies leading to inequalities between patient care in different geographical regions [14]. In the MS Barometer 2020, Hungary ranked in the middle range with 51 out of 100 points report [15] with relatively satisfying access to reimbursed therapies in 2018. According to the country-specific report [16], improved patient care may be achieved with the establishment of a national registry, guidelines on pediatric MS care, better access of patients to rehabilitation programs and symptomatic treatment. The report also suggested involvement of health care providers in the national reimbursement decision policies. In Hungary, treatments for pwMS approved by the European Medicines Agency (EMA) are usually available without reimbursement restrictions only by a delay of 2–4 years. During these years of restricted access, MS neurologists have to apply to the National Health Insurance Fund for reimbursement in each case. This is a time consuming process (mean evaluation time is 60 days) associated with a high administration load and high rejection rate. In Hungary, ocrelizumab is approved only for primary progressive MS, and individual applications are necessary for reimbursement in cases of relapsing MS. Of note, anti-CD20 therapies rank among the most effective therapies, especially if administered timely [17]. Lack of easily accessible anti-CD20 treatments for relapsing MS in Hungary complicates start with HET, escalation or switch strategies, especially in JCV positive patients with highly active or breakthrough disease (Fig. 1).

In 2018, the ECTRIMS guideline [19] was translated into Hungarian. The Hungarian national MS guideline was prepared in 2013, and a revised version is under development. The National Health Insurance Fund in Hungary uses a financial guideline with terms first and second line therapy, although these concepts are out of date in relation to the novel treatment strategies. Beside insufficient human resources and infrastructure [20], MS centers have to deal with restricted access to MS treatments in Hungary, which affects the quality of MS care.

The aim of our study is to highlight the required changes in Hungary and provide consensus recommendations on the treatment of relapsing MS by experts on a national level using the Delphi method.

## Methods

From November 2022 to February 2023, monthly online meetings of the Committee of the Hungarian Neuroimmunology Society addressed the preparation of a state-of-art national treatment recommendation using the Delphi method to achieve consensus [21]. Beside the Committee members of the Hungarian Neuroimmunology Society, MS experts from the largest national MS centers have also been involved in the rounds. The 17 consultant neurologists involved were covering the geographical distribution of Hungary and represented the staff of the largest MS centers in Hungary. The discussion about the recommendation was shaped by the 11 Steering Committee members of the Hungarian Neuroimmunology Society actually updating the Hungarian National MS Guidelines on monthly zoom meetings starting in November 2021 until November 2022, and the first Delphi round started in November 2022.

The participants could express their agreement on a 7-point scale (strongly agree, agree, somewhat agree, neither agree or disagree, somewhat disagree, disagree, strongly disagree). Consensus was achieved by agreements of > 80% on online distributed lists of open ended recommendations in 4 rounds.

## Results

### Recommendation 1

DMT should be offered to all patients with relapsing MS diagnosed based on the 2017 McDonald criteria [22]. Disease activity and prognostic factors should be considered in the choice of the disease modifying drug (Table 1). High-efficacy therapy (HET) should be offered to pwMS with high disease activity and poor prognostic factors (Table 2).

**Table 1:** Prognostic factors influencing MS disease outcome

	Poor prognosis
**Demographic and environmental factors**	• older age • male sex • non-White population • low vitamin D levels • smoking • comorbid conditions
**Clinical factors**	• polysymptomatic onset • early cognitive deficits • brainstem, cerebellar or spinal cord onset • primary progressive disease subtype • poor recovery from the 1st relapse • high relapse rate • short interval between the 1st and 2nd relapses • higher EDSS score at diagnosis
**Radiological factors**	• high T2 lesion number • high T2 lesion volume • presence of Gd-enhancing lesions • presence of infratentorial lesions • presence of spinal cord lesions • whole brain atrophy • grey matter atrophy
**Biomarkers**	• presence of IgG and IgM oligoclonal bands in the CSF • retinal nerve fiber layer thinning detected with optical coherence tomography

adapted from Rotstein 2019 [23]

abbreviations: CSF – cerebrospinal fluids, EDSS – expanded disability status scale, Ig – immunoglobulin

**Table 2:** DMTs in the treatment of MS

DMTs recommended for pwMS with moderate disease activity, 1st choice in escalation	dose and route of administration
interferon-beta-1b (Betaferon) interferon-beta-1a (Avonex) interferon-beta-1a (Rebif) PEG-interferon-beta-1a (Plegridy)	8 MIU s.c. every other day 30 ug i.m./week 44 ug s.c. 3 times/week 125 ug s.c. / 2 weeks
glatiramer acetate (Copaxone)	40 mg s.c. 3 times/week
teriflunomide (Aubagio)	14 mg/day
dimethyl fumarate (Tecfidera)	1st week 2 × 120 mg from the 2nd week 2 × 240 mg
diroximel fumarate (Vumerity)	1st week 2 × 231 mg from the 2nd week 2 × 462 mg
DMTs recommended for pwMS with high disease activity for escalation or as 1st choice*	**dose and route of administration**
fingolimod (Gilenya)	0.5 mg/day
siponimod (Mayzent)	Depending on the CYP2C9 genotype: Genotypes *1/*1, *1/2 or *2/*2 Days 1–2: 0.25 mg Day 3: 0.5 mg Day 4: 0.75 mg, then 1 mg/day
ozanomid (Zeposia)	Day 1–4: 0.23 mg Day 5–7: 0.46 mg, then 0.92 mg/day
ponezimod (Ponvory)	Days 1–2: 2 mg Days 3–4: 3 mg Days 5–6: 4 mg Day 7: 5 mg Day 8: 6 mg Day 9: 7 mg. Day 10: 8 mg Day 11: 9 mg, Day 12-14: 10mg, then 20mg/day
cladribine (Mavenclad)	3.5 mg/kg divided into 2 yearly courses 1 course = 2 cycles of 4-5days 23–27 days apart
natalizumab (Tysabri)	300 mg i.v./month or also available in 2 × 150 mg s.c./ month
alemtuzumab (Lemtrada)	2 cycles 12 months apart 1st cycle: 12 mg i.v. for 5 days 2nd cycle: 12 mg i.v. for 3 days
ocrelizumab (Ocrevus)	Day 1 and 15: 300 mg i.v., than every 6 months 600 mg i.v.
ofatumumab (Kesimpta)	20 mg s.c. Induction: Day 1, 7 and 14: 20 mg s.c., than every 4 weeks 20 mg s.c.

*Monoclonal antibody therapies (natalizumab, alemtuzumab, ocrelizumab and ofatumumab) are the most efficient DMTs. (based on the SmPCs of subsequent DMTs)

abbreviations: i.m. – intramuscular, i.v. – intravenous, s.c. – subcutaneous

Agreement achieved in Round 4 (88.24% agreed strongly, 5.88% agreed and 5.88% agreed somewhat).

### Recommendation 2

Patients on DMTs should be seen at least every 6 months by an MS specialist in an MS center and followed at least 6-monthly by expanded disability status scale (EDSS), and at least yearly by single digit modality test, 9-hole peg test and 25-feet walking test. For specific follow up on treatments, see the summary of product characteristics.

Agreement achieved in Round 2 (100% strongly agreed).

### Recommendation 3

Escalation of the therapy should be considered if the patient while on moderate efficacy therapy has a relapse and/or more than 2 new or enlarging T2 and/or Gd enhancing lesions on the follow-up annual MRI. Lateral switch is not recommended while on moderate efficacy therapy, unless the change of therapy is initiated due to side effects or intolerance or pregnancy planning.

After the 4th round 82.35% of the respondents have strongly agreed, and 17.65% agreed.

### Recommendation 4

In case of signs of radiological and/or clinical disease activity while on HET, change of therapy to another HET should be considered. For wash out time see Table 3.

**Table 3:** Wash out times for different DMTs

DMT	Wash-out time
GA	not necessary
IFN-beta	not necessary
DMF	not necessary, but normalization of lymphopenia or trend of normalization is recommended
teriflunomide	necessary, can be shortened by cholestyramine
fingolimod	≥ 4 weeks (but not exceeding 8 weeks)
natalizumab	≥ 4–8 weeks (but not exceeding 8 weeks)
alemtuzumab/cladribine	≥ 6–12 months
ocrelizumab/ofatumumab/rituximab	≥ 3–6 months

based on the data of Bigaut et al. 2021 [24]

abbreviations: DMT- disease modifying treatment, DMF – dimethyl fumarate, GA – glatiramer acetate, IFN- interferon,

In Round 2 the respondents answered with the following answers: 82.35% strongly agreed, 5.88 agreed.

### Recommendation 5

Regardless of age, de-escalation/discontinuation of treatment needs a cautious approach. In patients with progressive disease and EDSS ≥ 7, de-escalation/discontinuation can be considered. In non-progressive disease without clinical and MRI activity for years, de-escalation/discontinuation always needs individual assessment and is not routinely recommended. This should be based on the age, adverse events, potential risks of AEs, comorbidities and their treatment, radiological and clinical activity in the preceding 5 years, the ongoing DMT (caution particularly with natalizumab and fingolimod), and the effects/AEs of previous DMTs.

Agreement achieved in Round 2 (88.24% agreed strongly and 11.76% agreed).

### Recommendation 6

MS does not affect fertility; it has no adverse effect on pregnancy or neonatal outcomes. Women with family plans are advised to postpone the pregnancy until their disease is stable. Hormonal contraception is not contraindicated in MS. In case of IVF, GnRH agonists should be avoided. Both obstetric and neurological follow-up is recommended before, during and after the pregnancy. The risk of MS relapse decreases during pregnancy but increases in the first few months after birth. In case of relapses during pregnancy, indication of the corticosteroid treatment (500-1000 mg methylprednisolone (or equivalent) daily for 3–5 days) should be decided on a case-by-case basis. MRI without gadolinium contrast agent is permitted if necessary. MS professionals should consider the safety of a treatment during pregnancy when prescribing DMT to women of childbearing age. Administration of interferon-beta, glatiramer acetate, and natalizumab can be considered during pregnancy. Dimethyl fumarate and anti-CD20 therapies can be continued until pregnancy. For more details regarding DMT use in pregnancy and breastfeeding, see Table 4. During breastfeeding, short corticosteroid pulse therapy is permitted if necessary and should be decided individually.

**Table 4:** DMT use and family planning

DMT	Discontinuation before pregnancy	Use in pregnancy	Breastfeeding
glatiramer acetate	not necessary	compatible	compatible
INF-β	not necessary	compatible	compatible
dimethyl-fumarate / diroximel-fumarate	not necessary	contraindicated	contraindicated
teriflunomide	accelerated elimination	contraindicated	contraindicated
fingolimod	at least 2 months	contraindicated	contraindicated
siponimod	at least 10 days	contraindicated	contraindicated
ozanimod	at least 3 months	contraindicated	contraindicated
ponesimod	at least 1 week	contraindicated	contraindicated
cladribine	at least 6 months	contraindicated	7 days after the drug intake breastfeeding is compatible
natalizumab	not necessary	compatible in very active disease; stop at 30–34 GW; extended interval dosing (6 weeks)	compatible, no interval between infusion and next breastfeeding
ocrelizumab	until pregnancy	contraindicated	probably compatible, wait at least 4 h after infusion
ofatumumab	until pregnancy	contraindicated	compatible (except the first days after birth)
alemtuzumab	last dose 4 months before conception	contraindicated	probably compatible EMA: 4 months after the last infusion

based on SMPCs and Krysko et al. 2023 [25] abbreviations: EMA – European Medicine Agency, GW – gestational week

The responses in Round 2 were strongly agree: 82.24%, agree: 5.88% and somewhat disagree 5.88%.

### Recommendation 7

PwMS should be informed about optimal individualized immunization at diagnosis but latest before starting DMTs. Vaccines not containing live pathogens can be administered 3–5 days after short course of high dose corticosteroid treatment if the patient is in remission; live attenuated vaccines can be given 3 months after the steroid treatment. Ideally, vaccines not containing live pathogens should be administered at least 2–4 weeks before the start of the DMT (depending on the DMT), and the administration of the live attenuated vaccines (varicella, mumps, measles, rubella) should be completed 4–6 weeks before the start of the therapy. In case the initiation of DMT is urgent, one dosage from the varicella vaccine consisting of two dosages may be administered before the start of the therapy.

Agreement achieved in Round 2 (94.12% strongly agreed and 5.88% agreed).

### Recommendation 8

The clinical phenotype of late-onset MS may be different from early-onset MS, and is characterized by higher percentage of progressive course, pyramidal and cerebellar symptoms, while sensory symptoms are less prevalent. However, patients with late-onset MS reach disability later and prognostic factors may be similar. Therefore, the type of disease course may be more important than the age of onset. In aging MS patients the clinical and radiological activity declines, while comorbidities become more common. Although treatment choice is not different in active late-onset MS or aging MS, comorbidities may be important (e.g. risk of malignancy, lymphopenia, hypogammaglobulinemia, hypertension, infection and PML).

Agreement achieved in Round 2 (94.12% strongly agreed and 5,88% agreed).

## Conclusion

As our knowledge on MS pathomechanism is broadening, principles of treatment strategies change. Accordingly, treatment recommendations should be regularly updated. Several national guidelines are available with partial overlap. Definition of the disease activity and escalation strategy is somewhat different in the distinct guidelines (Table 5), probably reflecting adjustment to the different healthcare systems and additional factors at national level. Well-defined national consensus protocols may facilitate dialogue between policymakers and healthcare professionals and thus contribute to better patient care in the long run.

**Table 5:** Different approaches to therapy escalation

	relapse	MRI	progression (EDSS)	remarks
**Rio score (21)**	≥ 1	≥ 3 new T2 lesions	≥ 1 point	〈risk if the Rio score ≥ 2 points
**MAGNIMS score (22)**	≥ 1	≥ 3 new T2 lesions	-	〈risk if the MAGNIMS score ≥ 2 points
**NEDA** ^**3**^	≥ 1	≥ 1 new T2 lesions	≥ 1 point	better for positive predictive value
**EAN/ECTRIMS g.** ^**4**^	≥ 1	1 or 2 new T2 lesions	if ↑ (not defined)	combination of clinical and MRI parameters
**AAN guideline** ^**5**^	≥ 1	≥ 2 new T2 lesions	if ↑ (not defined)	tolerability and adherence
**Canadian recommendation** ^**6**^	≥ 1 major ≥ 2 minor	≥ 3 new T2 lesions	> 1 point	close monitoring if there is any sign of disease activity
**German recommendation** ^**7**^	≥ 1	≥ 2–3 new T2 lesions	0.5-1 point	in line with NEDA concept
**Croatian recommendation** ^**8**^	≥ 1	≥ 3 new T2 lesions	-	combination of clinical and MRI parameters

Rio 2009 [26], Sormani 2016 [27], Rotstein 2015 [28], Montalban 2018 [19], Rae-Grant 2018 [29], Freedman 2020 [11], Wiendl 2021 [12], Habek 2022 [13]

It is important to note that the consensus recommendations are not intended to replace critical thinking or individualization of the choice of DMTs and patient care.

## Electronic supplementary material

Below is the link to the electronic supplementary material.

Supplementary Material 1

Supplementary Material 2

## Data Availability

The datasets used and/or analysed during the current study are available from the corresponding author on reasonable request.

## Abbreviations

CSF: cerebrospinal fluids
DMT: disease modifying therapy
ECTRIMS: European Committee for Research and Treatment in Multiple Sclerosis
EDSS: expanded disability status scale
EMA: European Medicines Agency
GW: gestational week
Ig: immunoglobulin
i.m.: intramuscular
i.v.: intravenous
MS: multiple sclerosis
pwMS: people with MS
s.c.: subcutaneous
