# Supplementary material for: Treatment of relapsing multiple sclerosis in Hungary – consensus recommendation from the Hungarian neuroimmunology society
Source: Orphanet J Rare Dis. 2023 Jul 7;18:183. doi: 10.1186/s13023-023-02789-0 (PMC10329288; doi:10.1186/s13023-023-02789-0)
Supplement: Supplementary file 1 — Supplementary Material 1 [file 13023_2023_2789_MOESM1_ESM.docx]

**Professor Francesc Palau**

**editor-in-chief for Orphanet Journal of Rare Diseases**

Head of the Department of Genetic and Molecular Medicine

Director of the Pediatric Institute of Rare Diseases (IPER) of the Sant Joan de Déu Children’s Hospital

Barcelona,Spain

Dear Editor-in-Chief,

Please find the enclosed our manuscript entitled " **Treatment of relapsing multiple sclerosis in Hungary – consensus recommendation from the Hungarian Neuroimmunology Society”** by Rajda Cecilia et al.

As disease modifying treatment choices in multiple sclerosis are expanding, consensus recommendation can ease the clinicians’ decisions and preparation of national guidelines. We believe that our findings would appeal the readership of the Orphanet Journal of Rare Diseases.

We confirm that this manuscript has not been published elsewhere and is not under consideration by another journal.

All authors have approved the manuscript and agree with its submission to Orphanet Journal of Rare Diseases.

We look forward to hearing from you at your earliest convenience.

Sincerely yours,

Cecilia Rajda MD PhD

Department of Neurology, University of Szeged,

Semmelweis u.6. 6725 Szeged, Hungary

phone: +36 62 545348

fax: +36 62 545597

email: [rajda.cecilia@med.u-szeged.hu](mailto:rajda.cecilia@med.u-szeged.hu)
