# Supplementary material for: Treatment of relapsing multiple sclerosis in Hungary – consensus recommendation from the Hungarian neuroimmunology society
Source: Orphanet J Rare Dis. 2023 Jul 7;18:183. doi: 10.1186/s13023-023-02789-0 (PMC10329288; doi:10.1186/s13023-023-02789-0)
Supplement: Supplementary file 2 — Supplementary Material 2 [file 13023_2023_2789_MOESM2_ESM.docx]

**Point-wise rebuttal to the comments of the Reviewers**

We gratefully thank the Reviewers for their valuable remarks and constructive advice, and have shaped the manuscript accordingly.

**Reviewer #1**: This is is good paper, with importance in its field and practical recommendations to health professionals who treat relapsing multiple sclerosis (Hungary). A good consensus was achieved in the majority of recommendations.

We have changed the following sentences

“168 Agreement achieved in Round 2 (88.24% agreed strongly and 11.76% agreed)”

as suggested to:

168 Agreement achieved in Round 2 (88.24% agreed strongly and 11.76% agreed) (dot missing).

and

„206 disease course may be more important than the age of onset. In aging MS patients, the “

as suggested to

„206 disease course may be more important than the age of onset. In aging MS patients the..”.

**Reviewer #2:**

1.Regarding to the "Methods", I consider necessary to add how many other professionals participated (in addition to the 17 mentioned), the regions of the country to which they belong, and the inclusion criteria they used for this (how their expertise in the matter was measured). Also, it would give more solidity to the conclusions to explain more about the structure of the meetings, the discussion methodology that was used, its duration, etc.

We have added the suggested description to the Methods part (line 124-129):

Beside the Committee members of the Hungarian Neuroimmunology Society, MS experts from the largest national MS centers have also been involved in the rounds. The 17 consultant neurologists involved were covering the geographical distribution of Hungary and represented the staff of the largest MS centers in Hungary. The discussion about the recommendation was shaped by the 11 Steering Committee members of the Hungarian Neuroimmunology Society actually updating the Hungarian National MS Guidelines on monthly zoom meetings starting in November 2021 until November 2022, and the first Delphi round started in November 2022.

2. In recommendation 6 would it be necessary to indicate a maximum limit of corticosteroids?

Thank you for your valuable comment. We have added the recommended dose of the corticosteroids (line 182-184).

**In case of relapses during pregnancy, indication of the corticosteroid treatment (500-1000mg methylprednisolone (or equivalent) daily for 3-5 days) should be decided on a case-by-case basis.**

I consider it a very good job, with a high impact on the management of health policies. Its scope to professionals from all over the country gives it solidity and helps the effectiveness of public health management.
Thank you very much for the contribution. Congratulations.

We have corrected the reference No. 21.

The authors greatly appreciate the positive opinion of the Reviewers. We hope that the Editor and the Reviewers will find our edited manuscript worthy of publication.
